# Evaluation of the role of postoperative radiotherapy in locally invasive thymoma: A propensity-matched study based on the SEER database

**DOI:** 10.1371/journal.pone.0283192

**Published:** 2023-04-13

**Authors:** Li-mei Lin, Yi-min Li, Yun-xia Huang, Zong-kai Zhang, Ya-qing Dai, Jun Liu, Qin Lin

**Affiliations:** Department of Radiation Oncology, Xiamen Cancer Center, Xiamen Key Laboratory of Radiation Oncology, The First Affiliated Hospital of Xiamen University, School of Medicine, Xiamen University, Xiamen, Fujian, China; Tehran University of Medical Sciences, ISLAMIC REPUBLIC OF IRAN

## Abstract

**Objectives:**

No consensus was reached on the efficacy of postoperative radiotherapy (PORT) in locally invasive thymomas because of the rarity of the thymic epithelial and the variations of study results. Therefore, we aimed to explore the efficacy of PORT in locally invasive thymomas using the Surveillance, Epidemiology, and End Results (SEER) database.

**Methods:**

Patients diagnosed with thymomas from 2004 to 2016 were identified using the SEER database. Prognostic factors of cancer-specific survival (CSS) and overall survival (OS) were identified using univariate and multivariate Cox regression analyses.Propensity score matching (PSM) was performed to balance the baseline characteristics.

**Results:**

A total of 700 eligible patients were identified. After PSM, 262 paired patients were selected from the two groups, those who received or did not receive PORT. Receiving PORT improved CSS and OS before and after PSM. In the matched population, the multivariate analyses showed that tumour invasion into adjacent organs/structures and non-utilisation of PORT were independent poor prognostic factors for CSS, whereas age ≥62 years,tumour invasion into adjacent organs/structures, and non-utilisation of PORT were independently associated with poorer OS. The subgroup analysis revealed that PORT improved CSS and OS in Masaoka-Koga stage III thymoma, but showed no OS benefit in Masaoka-Koga stage IIB thymoma.

**Conclusion:**

Based on the SEER database, we found that PORT provides a significant survival benefit in Masaoka-Koga stage III thymoma with complete or incomplete resection. The role of PORT in thymoma requires further evaluation.

## Introduction

Thymoma is the most common tumour of the anterior mediastinum [1]. Its prevalence relatively low, with approximately 1.5 cases per million people in the United States [1]. The survival outcome of thymoma is largely dependent on the cancer stage, with 5-year overall survival (OS) ranging between 25% and 100% [2–5]. The Masaoka staging system, which was proposed in 1981, was considered to be a suitable predictor of prognosis in thymoma [6]; furthermore, Koga et al. introduced a modified Masaoka staging system of thymoma, and according to the Koga modifications stage II means invasion beyond the capsule [7].

Surgical resection is the mainstay treatment, and the extent of resection was proven to be an independent prognostic factor [8, 9]. The involvement of surrounding vital structures makes it difficult to have a complete resection, particularly for advanced-stage disease [10, 11]. Thus, postoperative radiotherapy (PORT) is often utilised in thymomas after incompletely resection to enhance tumour control [12, 13].

To date, the efficacy of PORT in thymoma remains unclear, and the utilisation of PORT is still left to the discretion of the attending surgeon or physician. Considering long-term survival, patients with stage I thymoma are not recommended to receive PORT [2, 14]. In stage II or III thymomas, therapeutic indications for PORT are still difficult to ascertain, and adjuvant radiation is frequently suggested in incompletely resected tumours [10, 13]. Some reports found no survival benefits in undergoing PORT after complete resection in early-stage thymomas [14–16], however, excellent tumour control has been observed with the use of PORT in some studies [10, 17]. Considering the limitations of the small retrospective studies, the role of PORT in stage II and III thymomas remains controversial.

Several studies have addressed the survival benefits of PORT using the Surveillance, Epidemiology, and End Results (SEER) database [18–21]. These population-based studies observed a significant difference in prognosis in favour of PORT, whereas it was not an independent predictor for survival. A recent meta-analysis including 4,746 patients recommended PORT for patients with stage II/III thymoma [22]. Indeed, some large international databases had been used to explore the efficacy of PORT. Analyses of the International Thymic Malignancies Interest Group Database by Rimner et al. [23] and the National Cancer Database by Jackson et al. [24] showed a significant survival benefit of PORT in stage II or III thymomas. In contrast, using the Japanese Association for Research on the Thymus database, Omasa et al found no survival benefit for PORT in these patients [25].

Our study aimed to evaluate the clinical implications of PORT in stages IIB and III thymomas using the SEER database. Patients with stage I disease were excluded for their excellent outcome and the limited benefit of PORT. Patients with stage IV disease were also excluded because many studies had showed a significant benefit of PORT in stage IV thymomas. Stage IIA was indistinguishable from stage I because the data on microscopic capsular invasion is not available in the SEER database. Therefore, in this study, we defined ‘locally invasive’ thymomas as the Masaoka stages IIB and III based on the SEER registry. We aimed to explore the efficacy of PORT in locally invasive thymomas using propensity score matching (PSM) of the SEER database.

## Materials and methods

### Ethics statement

This study was based on the SEER 18-Registry databases (1973–2015 data set), tracking nearly 28% of people in the United States. The SEER data are publicly available and do not require patient informed consent; therefore, institutional review approval was not required for our study. We extracted the dataset with the reference number 13027-Nov2018.

### Patients selection

**Fig 1:**
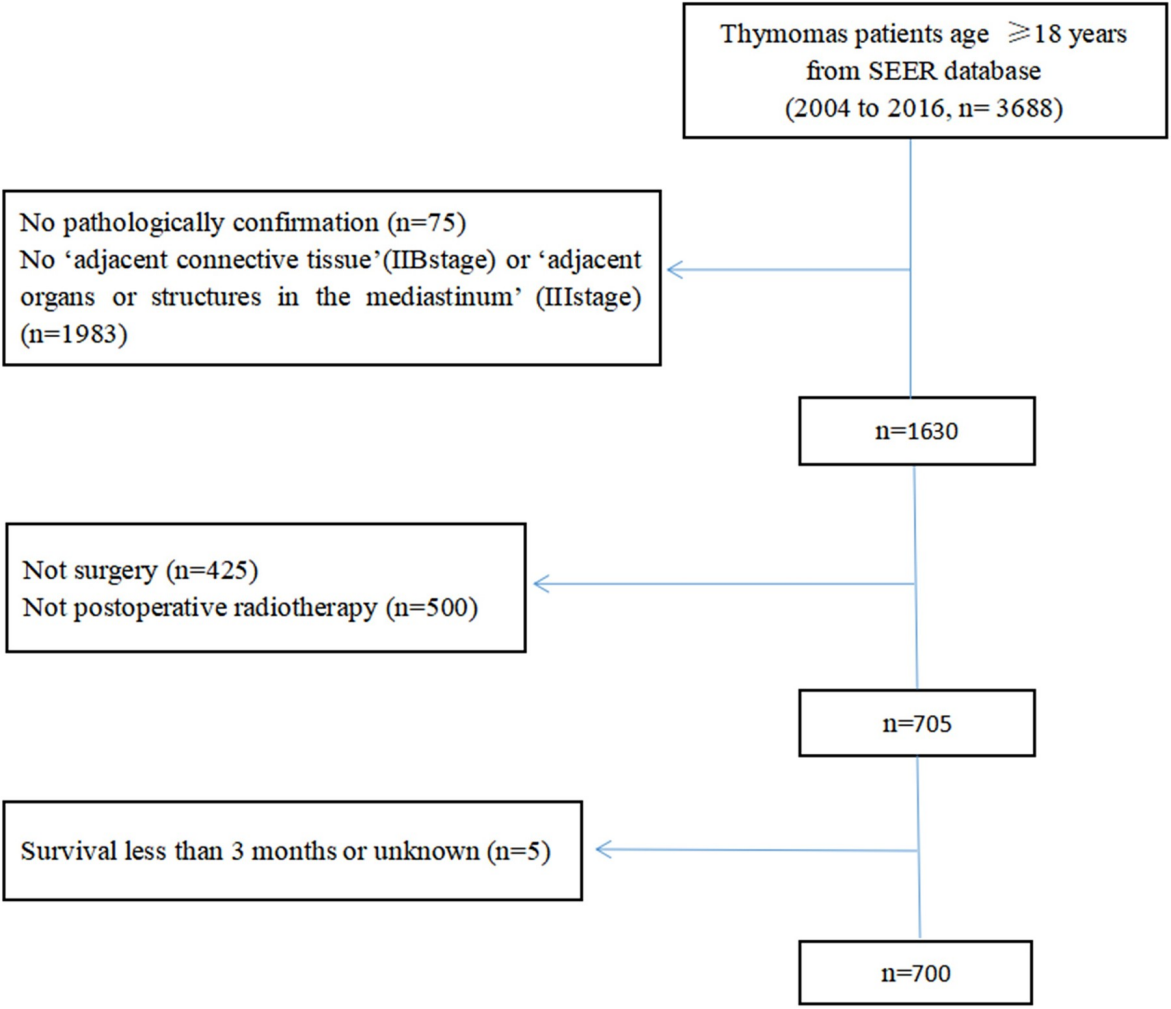


Primary cancer site and histology were identified using the International Classification of Disease for Oncology, third edition (ICD-O-3) of 8580, 8581, 8582, 8583, 8584, and 8585.A flowchart of patient selection is shown in Fig 1. The eligibility criteria were as follows: 1) diagnosis from 2004 to 2016, 2) patient aged ≥18 years, 3) survival duration ≥3 months, and 4) patients who underwent primary surgical resection with a postoperative status. The types of cancer-directed surgery in SEER included simple or partial resection, total resection, surgery stated to be ‘debulking’, and radical surgery. Simple or partial resection and total resection were identified according to the degree of macroscopic surgical removal. The radical resection was coded as partial or total removal of the primary site with an en bloc resection of other organs. Other information obtained from the database included sex, race, marital status, WHO classification, lymph node status, survival duration, and vital status. Data regarding margin status and chemotherapy used were not reported in the public-access SEER registry and therefore were not analysed in the study. The Masaoka-Koga classification was not clearly described in the SEER program, and we obtained patients’ Masaoka stage information from the variables of primary tumour extension, SEER historic stage, and lymph node status. The code ‘localized or organ-confined’ was in accordance with stage I/IIA, ‘adjacent connective tissue’ with stage IIB, and ‘adjacent organs or structures in the mediastinum’ with stage III. However, stage IIA could not be distinguished from stage I because the SEER database does not provide information about microscopic capsular invasion.

### Variable

The PSM model was based upon age, gender, race, marital status, adjacent connective tissue, lymph node status, extent of surgery, survival months, vital status, and cancer-specific death.

### Statistical analysis

All statistical analyses were performed using SPSS Statistics (version 22.0; IBM Corp). The Kaplan-Meier method and the log-rank test were used to compare OS and cancer-specific survival (CSS) in patients with or without PORT. The chi-square test was used to analyse the differences in covariates between the PORT and non-PORT groups. Using the Cox proportional hazards model, multivariate analysis was performed to identify the risk factors for OS and CSS in the matched population. To balance the baseline characteristics between the two groups, PSM was performed. Considering the variables of age, sex, race, marital status, lymph node status, primary tumour extent, and extent of surgery, the score was calculated using a logistic regression model. A 1:1 ratio matching between the two groups was performed to maximise the propensity score match based on the nearest neighbour method. For all tests, two-sided P-values <0.05 were considered as statistically significant.

## Results

### Baseline patient characteristics

From 2004 to 2016, a total of 700 patients diagnosed with thymoma were identified. The demographic and clinicopathological characteristicsare showed in Table 1. The median age was 54 years (19–88 years), with 349 (49.9%) men and 351 (50.1%) women. There were 293 (41.9%) patients with primary tumour invasion into the adjacent connective tissue and 407 (58.1%) with adjacent organs or structures invasion. The WHO classification type B3 had been proven to be a negative prognostic factor in the multivariate analysis [15] and there were 162 (23.2%) patients with type B3 in our study. A total of 189 (27.0%) patients were treated with radical surgery, 316 (45.1%) with total resection, 174 (24.9%) with simple or partial resection, and 21 (3.0%) with debulking surgery. A total of 420 patients received PORT.

**Table 1:** Patient characteristics (N = 700).

Variables	N	%
Age		
Median (range)	54 (19–88)	
<40	79	11.3
40–49	110	15.7
50–59	154	22.0
60–69	192	27.4
≥70	165	23.6
gender		
Men	349	49.9
Women	351	50.1
Race		
White	470	67.1
Black	91	13.0
Others	126	18.0
Unknown	13	1.9
Marital status		
Married	439	62.7
Not married	243	34.7
Unknown	18	2.56
Tumor extent		
Adjacent connective tissue	293	41.9
Adjacent organs or structures	407	58.1
WHO classification		
Not otherwise specified	143	20.4
Type A	59	8.4
Type AB	121	17.3
Type B1	101	14.4
Type B2	114	16.3
Type B3	162	23.2
Lymph node status		
Negative	633	90.4
Positive	31	4.4
Unknown	36	5.2
Extent of surgery		
Radical surgery	189	27.0
Total resection	316	45.1
Simple or partial resection	174	24.9
Debulking surgery	21	3.0
PORT		
Yes	420	60.0
No	280	40.0

Abbreviations: WHO, World Health Organization; PORT, postoperative radiotherapy.

### Survival before PSM

**Fig 2:**
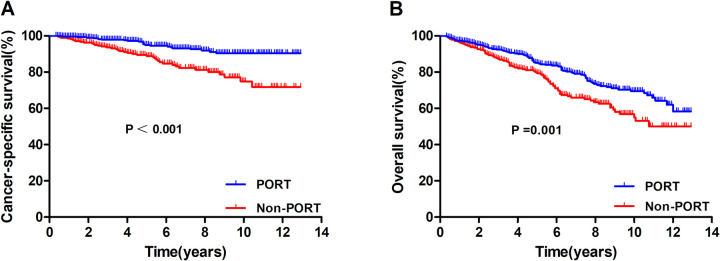
Comparison of cancer-specific survival (A) and overall survival (B) according to the receipt of postoperative radiotherapy before propensity score matching.

In the entire cohort, the 5 and 10-year CSS rates were 92.3% and 84.6%, respectively, and the 5 and 10-year OS rates were 82.1% and 63.9%, respectively. Survival outcomes for those who underwent PORT and those who did not are presented in Fig 2. There were statistically significant differences in CSS (P < 0.001) and OS (P = 0.001) in favour of the PORT group. The 10-year CSS and OS of the PORT group were 90.4% and 69.5%, respectively, and the 10-year CSS and OS of the non-PORT group were 74.9%% and 55.0%, respectively.

### Prognostic analysis after PSM

**Fig 3:**
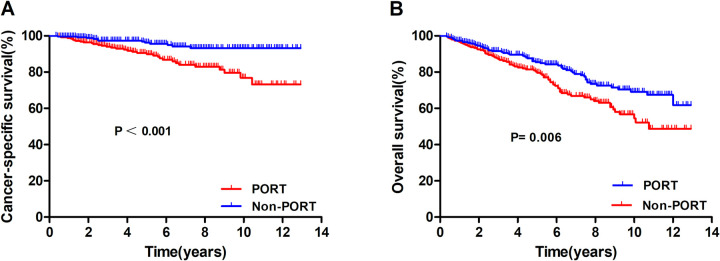
Comparison of cancer-specific survival (A) and overall survival (B) according to the receipt of postoperative radiotherapy after propensity score matching.

A total of 262 patient-pairs were completely matched. Table 2 presents the patient characteristics before and after PSM, with no significant difference between the matched groups. Fig 3A and 3B show the survival of CSS and OS in the propensity-matched cohort. There was a significant difference in the 10-year CSS and OS according to the receipt of PORT (CSS: 93.2% vs. 76.9%, P < 0.001; OS: 69.1% vs. 54.5%, P = 0.006).

**Table 2:** Patient, tumor, and treatment characteristics before and after propensity score matching.

Variables	Before Propensity Score Matching			After Propensity Score Matching		
	PORT (+) (N = 420)	PORT (−) (N = 280)	P	PORT (+) (N = 262)	PORT (−) (N = 262)	P
Age						
<40	47(11.2)	32(11.4)	0.009**	25(9.5)	29(11.1)	0.19
40–49	69(16.4)	41(14.6)		33(12.6)	38(14.5)	
50–59	106(25.2)	48(17.2)		64(24.4)	46(17.5)	
60–69	117(27.9)	75(26.8)		80(30.6)	72(27.5)	
≥70	81(19.3)	84(30.0)		60(22.9)	77(29.4)	
gender						
Men	219(52.1)	130(46.4)	0.139	127(48.5)	123(46.9)	0.726
Women	201(47.9)	150(53.6)		135(51.5)	139(53.1)	
Race						
White	282(67.2)	188(67.1)	0.804	181(69.1)	182(69.5)	0.587
Black	51(12.1)	40(14.3)		30(11.4)	36(13.7)	
Others	79(18.8)	47(16.8)		51(19.5)	44(16.8)	
Unknown	8(1.9)	5(1.8)				
Marital status						
Married	262(62.4)	177(63.2)	0.974	150(57.3)	152(58.0)	0.860
Not married	147(35.0)	96(34.3)		112(42.7)	110(42.0)	
Unknown	11(2.6)	7(2.5)				
Tumor extent						
Adjacent connective tissue	176(41.9)	117(41.8)	0.975	129(49.2)	111(42.4)	0.115
Adjacent organs or structures	244(58.1)	163(58.2)		133(50.8)	151(57.6)	
Lymph node status						
Negative	379(90.3)	254(90.7)	0.978	247(94.3)	252(96.2)	0.305
Positive	19(4.5)	12(4.3)		15(5.7)	10(3.8)	
Unknown	22(5.2)	14(5.0)				
Extent of surgery						
Radical surgery	119(28.3)	70(25.0)	0.047	69(26.3)	64(24.5)	0.157
Total resection	186(44.3)	130(46.4)		116(44.3)	125(47.7)	
Simple or partial resection	97(23.1)	77(27.5)		66(25.2)	70(26.7)	
Debulking surgery	18(4.3)	3(1.1)		11(4.2)	3(1.1)	

Abbreviations: PORT, postoperative radiotherapy.

In the univariate analyses (Table 3), tumour extension (P < 0.001) and utilisation of PORT (P = 0.001) were significant prognostic factors for CSS, whereas age (P < 0.001), tumour extension (P < 0.001), and utilisation of PORT (P < 0.001) were significant prognostic factors for OS.

**Table 3:** Univariate Cox regression analysis of prognostic factors in matched population.

Variables		CSS			OS	
	HR	95%CI	P	HR	95%CI	P
Age						
<62						
≥62	1.502	0.825–2.734	0.183	2.843	1.989–4.063	<0.001***
gender						
Men/Women	1.542	0.847–2.809	0.156	1.340	0.937–1.918	0.109
Race						
White	1			1		
Black	0.693	0.327–1.469	0.338	1.109	0.650–1.892	0.705
Others	0.980	0.365–2.633	0.968	1.163	0.720–1.879	0.537
Marital status						
Married/Not married	1.314	0.658–2.147	0.208	1.142	0.613–1.793	0.614
Tumor extent						
Adjacent connective tissue	1			1		
Adjacent organs or structures	4.130	1.922–8.874	<0.001***	2.271	1.534–3.362	<0.001***
WHO classification						
nontype B3/type B3	1.177	0.754–1.836	0.474	1.039	0.794–1.359	0.780
Lymph node status						
Negative/Positive	0.392	0.054–2.847	0.355	0.731	0.298–1.789	0.492
Extent of surgery						
Radical surgery	1			1		
Total resection	0.966	0.438–2.130	0.932	0.787	0.513–1.209	0.275
Simple or partial resection	1.851	0.839–4.082	0.127	1.363	0.878–2.117	0.167
Debulking surgery	3.248	0.893–9.816	0.074	1.215	0.433–3.413	0.711
PORT						
Yes/No	2.860	1.723–4.747	<0.001***	1.602	1.188–2.159	0.002**

Abbreviations: HR, hazard ratio; CI, confidence interval; CSS, cancer-specific survival; OS, overall survival; WHO, World Health Organization; PORT, postoperative radiotherapy.

Multivariate analysis (Table 4) incorporating covariates that were significant in the univariate analysis showed that tumour invasion into adjacent organs/structures (hazard ratio [HR], 3.957; 95% confidence interval [CI], 1.840−8.507; P <0.001) and non-utilisation of PORT (HR, 3.077; 95% CI, 1.585−5.974; P = 0.001) were independent poor prognostic factors for CSS, whereas age ≥ 62 years (HR, 2.805; 95% CI, 1.957−4.019; P <0.001), tumour invasion into adjacent organs/structures (HR, 2.326; 95% CI, 1.569−3.446; P <0.001), and non-utilisation of PORT (HR, 1.692; 95% CI, 1.171−2.445; P = 0.005) were independently associated with poorer OS (Table 4).

**Table 4:** Multivariate Cox regression analysis of prognostic factors in matched population.

Variables		CSS			OS	
	HR	95%CI	P	HR	95%CI	P
Age						
<62						
≥62				2.805	1.957–4.019	<0.001***
Tumor extent						
Adjacent connective tissue	1		1			
Adjacent organs or structures	3.957	1.840–8.507	<0.001***	2.326	1.569–3.446	<0.001***
PORT						
Yes/ No	3.077	1.585–5.974	0.001***	1.692	1.171–2.445	0.005**

Abbreviations: HR, hazard ratio; CI, confidence interval; CSS, cancer-specific survival; OS, overall survival; PORT, postoperative radiotherapy.

### Subgroup analysis

**Fig 4:**
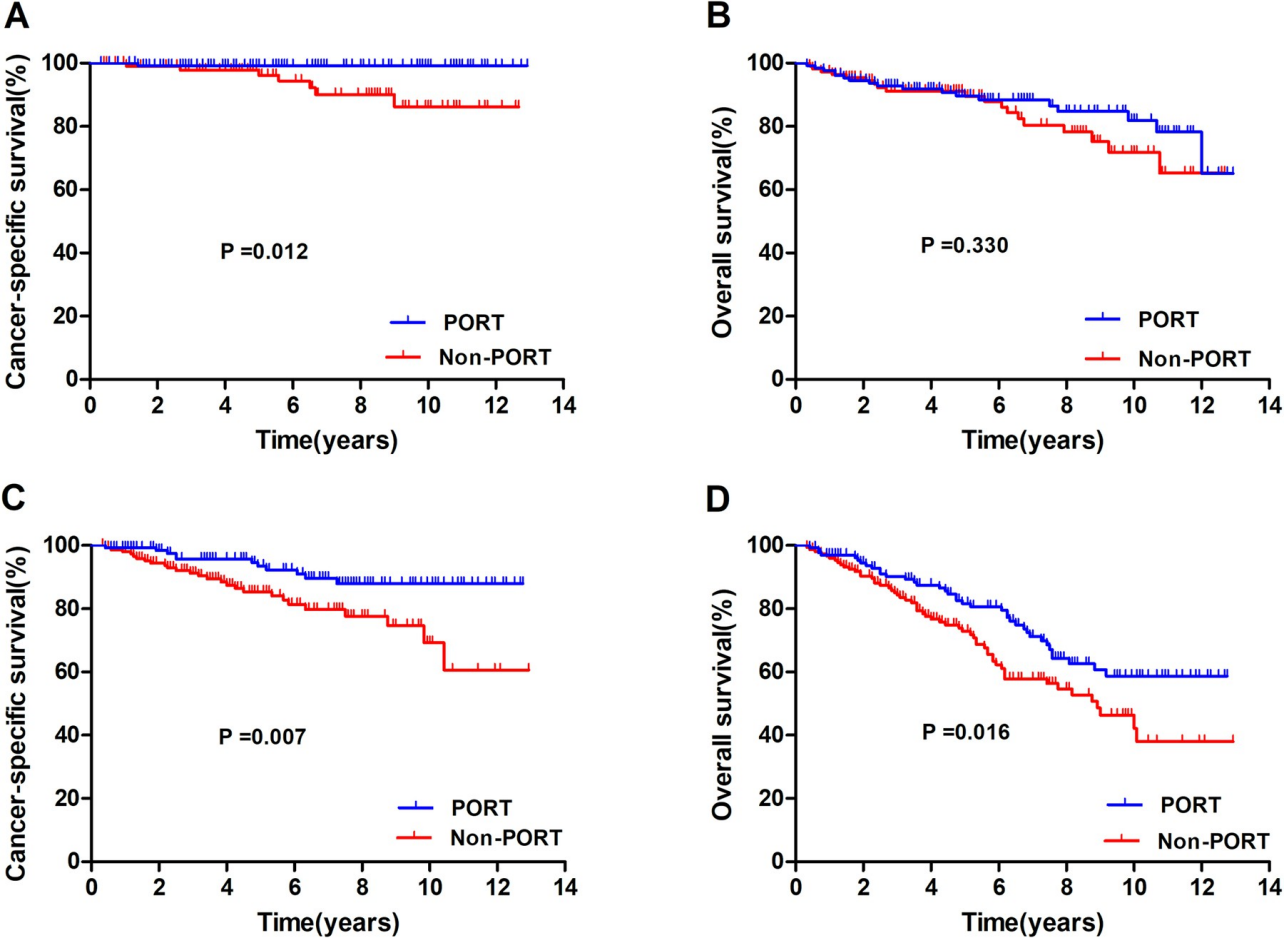
Cancer-specific and overall survival of the stage IIB (A and B) and stage III (C and D) with or without postoperative radiotherapy.

To identify potential patients who might benefit from PORT, a subgroup analysis was performed. Fig 4 shows the survival curve of CSS and OS in the subgroup analyses of patients with stages IIB and III thymoma. In stage IIB thymomas (Fig 4A), PORT showed a significant correlation with CSS (P = 0.012), but not with OS (P = 0.330). In stage III thymomas (Fig 4B), PORT showed a significant correlation with better CSS (P = 0.007) and OS (P = 0.016).

## Discussion

The decision on the optimal treatment for patients with thymomas is very important for physicians. However, because of the rarity of thymic epithelialtumorsand the variations of study results, the appropriate treatment for locally invasive thymomas remained unclear [26–28]. PORT is frequently performed for thymoma patients in clinical practice; however, no consensus was reached on the efficacy of PORT in this disease. The prognostic influence of PORT in stage I disease is considered to be limited because of its excellent outcome [14, 29]. The present study assessed the efficacy of PORT in stages IIB and III thymomas using the population-based SEER database. Adjuvant radiotherapy (RT) showed a significant correlation with OS and CSS of localised thymomas before and after PSM in our study.

In this study, PORT improved CSS and OS in stage IIB/III thymomas before and after PSM. Multivariate analysis showed that age, tumour extensions, and PORT were independently associated with OS, whereas tumour extensions and PORT were independently associated with CSS. The subgroup analysis showed that PORT improved CSS and OS in Masaoka-Koga stage III thymoma, but showed no OS benefit in Masaoka-Kogastage IIB thymoma.

The optimal utilisation of PORT in stage II thymomas is unclear, and several studies have indicated few survival benefits of PORT in these patients. Berman et al. showed no significant benefit of PORT in decreasing local recurrence rates in stage II thymoma with complete resection. The recurrence rate in patients who did not undergo PORT was 8.3% (2/24), and no recurrence (0/38) was found in those who underwent PORT (P = 0.15) [16]. The proportion of recurrence (8.3%) was quite low, making it difficult to detect a statistically significant difference in adjuvant RT. Using the British Columbia Cancer Agency Registry, another population-based analysis showed no significant OS benefit or freedom from recurrence in stage II thymoma after PORT [30]. Furthermore, Chang et al. reported that adjuvant RT described no significant correlation with disease-free survival(DFS) in stage II thymoma (P = 0.11) [31]. Similarly, a propensity-matched analysis from the SEER registry, including 592 cases with stages III−IV thymoma, showed statistically significant differences in disease-specific survival (DSS) and OS for PORT. However, no survival benefit of PORT was found in stage IIB thymomas which was similar with our study [32]. In contrast, Jackson et al. reported a significant OS benefit of PORT in stage II thymoma [24]. It may be explained that in the study by Jackson et al. more patients in the PORT group had positive surgical margin status than the non-PORT group (40.1% vs. 17.6%); however, the SEERS data of the current study and the study by Lim et al. are poor, given the lack of data on marginal status and preoperative chemotherapy.

Approximately30% of patients with stage III thymoma were at a risk of relapse [33]. The majority of recurrences were observed at the pleura (54%) and tumour bed (23%), even after complete resection [34]. This pattern of failure encourages clinicians to use PORT to control the tumour bed. Although widely used, the efficacy of PORT in stage III thymomas was still controversial. In a large population-based study, PORT improved the DSS and OS of stage III thymoma patients (n = 499) on univariate analysis [21], however, patients who received PORT were younger, and a larger proportion of them had undergone debulking surgery, which might have led to a provider bias in making treatment decisions. A multi-institutional propensity score-matched analysis from the European database concluded that adjuvant therapy was beneficial for stage III thymomas, especially for stage pT3 thymomas with a tumor size smaller than 5 cm [35]. Similarly, Liao et al. analysed 130 patients with completely resected stage III disease, 57% of whom received PORT, and found a trend of superior DSS in the PORT group. In multivariate analyses, PORT was proven to be an independent prognostic factor of OS [36]. A large-scale analysis from the multi-institutional national database in Japan concluded that PORT did not improve relapse-free survival or OS in stage III thymoma, which contradicts our findings [25]. This may be because the baseline covariates, especially the surgical modalities, were different between the Japanese and our cohort. Nearly 100% of the population in the Japanese cohort received complete resection, however only 72.1% in our study received complete resection. On the other hand, the Japanese study disregard PSM that we performed to balancethe baseline characteristics.

To date, there have been several SEER-based analyses of thymoma [18–20]. Patel et al. and Forqueret al. showed an OS benefit of PORT in stages II−III thymoma using the SEER database [18, 19]. Fernandes et al. showed a particular benefit of PORT for patients with stage III or IV thymoma in a univariate analysis; however, the statistical significance was eliminated in the multivariate analysis [20]. Additionally, the patients included in previous studies were diagnosed as early as the 1970s [18–20] whereas our analysis included patients diagnosed with thymoma from 2004 to 2016, which were mostly treated with modern RT techniques. In fact, the high-quality modern RT techniques had been demonstrated to reduce relapse rates and improve survival compared with conventional RT [37]. However, further research is needed to evaluate the survival benefit of PORT in the modern RT era.

Meta-analyses are another important ways to explorethe efficacy of PORT in thymoma. A meta-analysis by Zhou et al. showed that adjuvant RT had no survival benefit in the entire cohort of completely resected thymoma, but resulted in a favourable prognosis for OS in stage II/III thymoma [38]. Another meta-analysis byLim et al. analysis on 1724 patients and observed the survival advantages of PORT in stages III−IV thymomas, but not in stage II disease [39]. In contrast, Ma et al. found no benefit of PORT on recurrent risk in completely resected stage II or III thymomas [40]. These meta-analysesshow the difficulty in evaluating a prognosticrole of PORT in thymoma.

This study had several limitations. First, information on surgical margin status was not included in the registry, which may lead to inconsistent survival analysis. Second, there was no central review to confirm stage and histotype, which might lead to the variability in diagnosis among different pathologists. Third, the effect of chemotherapy was not analysed in our study because the information was not available in the SEER database. In addition to the chemotherapy data, details of the RT treatment (such as total radiation dose, daily fraction, and radiation techniques) were not included in the SEER database. Fourth, although we performed the PSM to reduce the effects of selection bias in performing PORT, the possibility of unpredictable confounders cannot be fully avoided for the limitations existing in any retrospective study. Finally, the SEER database contains little information to guide the analysis of why PORT was performed in each patient. Despite these limitations, the SEER database is still a useful tool to fill knowledge gaps and solve difficult questions, for example, in the management of thymomas. As a relatively rare disease, it was quite difficult to recruit a sufficient sample within a short time to perform a prospective randomised controlled trial. The SEER database provides a large number of patients treated in different institutions, improving the pool’s heterogeneity and allowing a long-term follow-up.

## Conclusion

According to the guidelinesof the European Society for Medical Oncology (EMSO) [41], PORT is recommended following complete resection of stage III thymoma (grade of recommendation IV, level of evidence B) because of the higher risk of disease recurrence and should be considered in thymoma with extensive transcapsular invasion (stage IIB) and aggressive histology, such as types B2 and B3 (grade of recommendation IV, level of evidence C). The present study demonstrated that PORT improved CSS and OS in Masaoka-Koga stage III thymoma, and seem to show no OS benefit in stage IIB thymoma. However, due to the inherent limitation of this study which was observational, the results can not imply rejection of PORT for stage IIBthymoma. Further prospective trials are warranted to confirm these results.

## Supporting information

S1 Table Patient characteristics before and after propensity score matching with effect size. (DOCX)

## References

[1] Engels EA. Epidemiology of thymoma and associated malignancies. Journal of thoracic oncology: official publication of the International Association for the Study of Lung Cancer. 2010;5(10 Suppl 4):S260–5. Epub 2010/10/05. doi: 10.1097/JTO.0b013e3181f1f62d ; PubMed Central PMCID: PMC2951303. 20859116 PMC2951303

[2] Kondo K, Monden Y. Therapy for thymic epithelial tumors: a clinical study of 1,320 patients from Japan. The Annals of thoracic surgery. 2003;76(3):878–84; discussion 84–5. Epub 2003/09/10. doi: 10.1016/s0003-4975(03)00555-1 . 12963221

[3] Venuta F, Anile M, Diso D, Vitolo D, Rendina EA, De Giacomo T, et al. Thymoma and thymic carcinoma. European journal of cardio-thoracic surgery: official journal of the European Association for Cardio-thoracic Surgery. 2010;37(1):13–25. Epub 2009/07/21. doi: 10.1016/j.ejcts.2009.05.038 . 19615917

[4] Guerrera F, Rendina EA, Venuta F, Margaritora S, Ciccone AM, Novellis P, et al. Does the World Health Organization histological classification predict outcomes after thymomectomy? Results of a multicentre study on 750 patients. European journal of cardio-thoracic surgery: official journal of the European Association for Cardio-thoracic Surgery. 2015;48(1):48–54. Epub 2014/09/24. doi: 10.1093/ejcts/ezu368 . 25246487

[5] Scorsetti M, Leo F, Trama A, D’Angelillo R, Serpico D, Macerelli M, et al. Thymoma and thymic carcinomas. Critical reviews in oncology/hematology. 2016;99:332–50. Epub 2016/01/29. doi: 10.1016/j.critrevonc.2016.01.012 . 26818050

[6] Masaoka A, Monden Y, Nakahara K, Tanioka T. Follow-up study of thymomas with special reference to their clinical stages. Cancer. 1981;48(11):2485–92. Epub 1981/12/01. doi: 10.1002/1097-0142(19811201)48:11&lt;2485::aid-cncr2820481123&gt;3.0.co;2-r . 7296496

[7] Koga K, Matsuno Y, Noguchi M, Mukai K, Asamura H, Goya T, et al. A review of 79 thymomas: modification of staging system and reappraisal of conventional division into invasive and non-invasive thymoma. Pathology international. 1994;44(5):359–67. Epub 1994/05/01. doi: 10.1111/j.1440-1827.1994.tb02936.x . 8044305

[8] Maggi G, Casadio C, Cavallo A, Cianci R, Molinatti M, Ruffini E. Thymoma: results of 241 operated cases. The Annals of thoracic surgery. 1991;51(1):152–6. Epub 1991/01/01. doi: 10.1016/0003-4975(91)90478-9 . 1985561

[9] Blumberg D, Port JL, Weksler B, Delgado R, Rosai J, Bains MS, et al. Thymoma: a multivariate analysis of factors predicting survival. The Annals of thoracic surgery. 1995;60(4):908–13; discussion 14. Epub 1995/10/01. doi: 10.1016/0003-4975(95)00669-c . 7574993

[10] Curran WJ Jr, Kornstein MJ, Brooks JJ, Turrisi AT 3rd., Invasive thymoma: the role of mediastinal irradiation following complete or incomplete surgical resection. Journal of clinical oncology: official journal of the American Society of Clinical Oncology. 1988;6(11):1722–7. Epub 1988/11/01. doi: 10.1200/jco.1988.6.11.1722 . 3183702

[11] Häfner MF, Roeder F, Sterzing F, Krug D, Koerber SA, Kappes J, et al. Postoperative radiotherapy of patients with thymic epithelial tumors (TET): a retrospective analysis of outcome and toxicity. Strahlentherapie und Onkologie: Organ der Deutschen Rontgengesellschaft [et al]. 2015;191(2):133–40. Epub 2014/08/27. doi: 10.1007/s00066-014-0740-z . 25156510

[12] Curran W, Kornstein M, Brooks J, Turrisi A. Invasive thymoma: the role of mediastinal irradiation following complete or incomplete surgical resection. Journal of clinical oncology: official journal of the American Society of Clinical Oncology. 1988;6(11):1722–7. doi: 10.1200/JCO.1988.6.11.1722 . 3183702

[13] Jackson MA, Ball DL. Post-operative radiotherapy in invasive thymoma. Radiotherapy and oncology: journal of the European Society for Therapeutic Radiology and Oncology. 1991;21(2):77–82. Epub 1991/06/01. doi: 10.1016/0167-8140(91)90078-u . 1866468

[14] Utsumi T, Shiono H, Kadota Y, Matsumura A, Maeda H, Ohta M, et al. Postoperative radiation therapy after complete resection of thymoma has little impact on survival. Cancer. 2009;115(23):5413–20. Epub 2009/08/18. doi: 10.1002/cncr.24618 . 19685527

[15] Chen YD, Feng QF, Lu HZ, Mao YS, Zhou ZM, Ou GF, et al. Role of adjuvant radiotherapy for stage II thymoma after complete tumor resection. International journal of radiation oncology, biology, physics. 2010;78(5):1400–6. Epub 2010/04/10. doi: 10.1016/j.ijrobp.2009.09.066 . 20378264

[16] Berman AT, Litzky L, Livolsi V, Singhal S, Kucharczuk JC, Cooper JD, et al. Adjuvant radiotherapy for completely resected stage 2 thymoma. Cancer. 2011;117(15):3502–8. Epub 2011/02/03. doi: 10.1002/cncr.25851 . 21287527

[17] Ogawa K, Uno T, Toita T, Onishi H, Yoshida H, Kakinohana Y, et al. Postoperative radiotherapy for patients with completely resected thymoma: a multi-institutional, retrospective review of 103 patients. Cancer. 2002;94(5):1405–13. Epub 2002/03/29. doi: 10.1002/cncr.10373 . 11920495

[18] Patel S, Macdonald OK, Nagda S, Bittner N, Suntharalingam M. Evaluation of the role of radiation therapy in the management of malignant thymoma. International journal of radiation oncology, biology, physics. 2012;82(5):1797–801. Epub 2011/05/21. doi: 10.1016/j.ijrobp.2011.03.010 . 21596484

[19] Forquer JA, Rong N, Fakiris AJ, Loehrer PJ Sr, Johnstone PA. Postoperative radiotherapy after surgical resection of thymoma: differing roles in localized and regional disease. International journal of radiation oncology, biology, physics. 2010;76(2):440–5. Epub 2009/05/12. doi: 10.1016/j.ijrobp.2009.02.016 . 19427738

[20] Fernandes AT, Shinohara ET, Guo M, Mitra N, Wilson LD, Rengan R, et al. The role of radiation therapy in malignant thymoma: a Surveillance, Epidemiology, and End Results database analysis. Journal of thoracic oncology: official publication of the International Association for the Study of Lung Cancer. 2010;5(9):1454–60. Epub 2010/07/24. doi: 10.1097/JTO.0b013e3181e8f345 . 20651611

[21] Weksler B, Shende M, Nason KS, Gallagher A, Ferson PF, Pennathur A. The role of adjuvant radiation therapy for resected stage III thymoma: a population-based study. The Annals of thoracic surgery. 2012;93(6):1822–8; discussion 8–9. Epub 2012/05/04. doi: 10.1016/j.athoracsur.2012.03.004 . 22551847

[22] Tateishi Y, Horita N, Namkoong H, Enomoto T, Takeda A, Kaneko T. Postoperative Radiotherapy for Completely Resected Masaoka/Masaoka-Koga Stage II/III Thymoma Improves Overall Survival: An Updated Meta-Analysis of 4746 Patients. Journal of thoracic oncology: official publication of the International Association for the Study of Lung Cancer. 2021;16(4):677–85. Epub 2021/01/31. doi: 10.1016/j.jtho.2020.12.023 . 33515812

[23] Rimner A, Yao X, Huang J, Antonicelli A, Ahmad U, Korst RJ, et al. Postoperative Radiation Therapy Is Associated with Longer Overall Survival in Completely Resected Stage II and III Thymoma-An Analysis of the International Thymic Malignancies Interest Group Retrospective Database. Journal of thoracic oncology: official publication of the International Association for the Study of Lung Cancer. 2016;11(10):1785–92. Epub 2016/06/28. doi: 10.1016/j.jtho.2016.06.011 ; Central PMCID: PMC5257334. 27346413 PMC5257334

[24] Jackson MW, Palma DA, Camidge DR, Jones BL, Robin TP, Sher DJ, et al. The Impact of Postoperative Radiotherapy for Thymoma and Thymic Carcinoma. Journal of thoracic oncology: official publication of the International Association for the Study of Lung Cancer. 2017;12(4):734–44. Epub 2017/01/28. doi: 10.1016/j.jtho.2017.01.002 . 28126540

[25] Omasa M, Date H, Sozu T, Sato T, Nagai K, Yokoi K, et al. Postoperative radiotherapy is effective for thymic carcinoma but not for thymoma in stage II and III thymic epithelial tumors: the Japanese Association for Research on the Thymus Database Study. Cancer. 2015;121(7):1008–16. Epub 2015/01/08. doi: 10.1002/cncr.29166 . 25565590

[26] Mangi A, Wright C, Allan J, Wain J, Donahue D, Grillo H, et al. Adjuvant radiation therapy for stage II thymoma. The Annals of thoracic surgery. 2002;74(4):1033–7. doi: 10.1016/s0003-4975(02)03828-6 . 12400741

[27] Mangi A, Wain J, Donahue D, Grillo H, Mathisen D, Wright C. Adjuvant radiation of stage III thymoma: is it necessary? The Annals of thoracic surgery. 2005;79(6):1834–9. doi: 10.1016/j.athoracsur.2004.12.051 . 15919266

[28] Korst R, Kansler A, Christos P, Mandal S. Adjuvant radiotherapy for thymic epithelial tumors: a systematic review and meta-analysis. The Annals of thoracic surgery. 2009;87(5):1641–7. doi: 10.1016/j.athoracsur.2008.11.022 . 19379938

[29] Mou H, Liao Q, Hou X, Chen T, Zhu Y. Clinical characteristics, risk factors, and outcomes after adjuvant radiotherapy for patients with thymoma in the United States: analysis of the Surveillance, Epidemiology, and End Results (SEER) Registry (1988–2013). International journal of radiation biology. 2018;94(5):495–502. Epub 2018/03/20. doi: 10.1080/09553002.2018.1454618 . 29553917

[30] Mariano C, Ionescu DN, Cheung WY, Ali RH, Laskin J, Evans K, et al. Thymoma: a population-based study of the management and outcomes for the province of British Columbia. Journal of thoracic oncology: official publication of the International Association for the Study of Lung Cancer. 2013;8(1):109–17. Epub 2012/12/18. doi: 10.1097/JTO.0b013e318276241c . 23242441

[31] Chang JH, Kim HJ, Wu HG, Kim JH, Kim YT. Postoperative radiotherapy for completely resected stage II or III thymoma. Journal of thoracic oncology: official publication of the International Association for the Study of Lung Cancer. 2011;6(7):1282–6. Epub 2011/06/07. doi: 10.1097/JTO.0b013e31821f9662 . 21642871

[32] Lim YJ, Kim HJ, Wu HG. Role of Postoperative Radiotherapy in Nonlocalized Thymoma: Propensity-Matched Analysis of Surveillance, Epidemiology, and End Results Database. Journal of thoracic oncology: official publication of the International Association for the Study of Lung Cancer. 2015;10(9):1357–63. Epub 2015/08/19. doi: 10.1097/JTO.0000000000000619 . 26280586

[33] Ruffini E, Mancuso M, Oliaro A, Casadio C, Cavallo A, Cianci R, et al. Recurrence of thymoma: analysis of clinicopathologic features, treatment, and outcome. The Journal of thoracic and cardiovascular surgery. 1997;113(1):55–63. doi: 10.1016/S0022-5223(97)70399-4 . 9011702

[34] Myojin M, Choi N, Wright C, Wain J, Harris N, Hug E, et al. Stage III thymoma: pattern of failure after surgery and postoperative radiotherapy and its implication for future study. International journal of radiation oncology, biology, physics. 2000;46(4):927–33. doi: 10.1016/s0360-3016(99)00514-3 . 10705015

[35] Leuzzi G, Rocco G, Ruffini E, Sperduti I, Detterbeck F, Weder W, et al. Multimodality therapy for locally advanced thymomas: A propensity score-matched cohort study from the European Society of Thoracic Surgeons Database. The Journal of thoracic and cardiovascular surgery. 2016;151(1):47–57.e1. Epub 2015/09/26. doi: 10.1016/j.jtcvs.2015.08.034 . 26403869

[36] Liao J, Liu T, Zhang H, Cai F, Chen J, Dang J. The role of postoperative radiation therapy for completely resected stage III thymoma and effect of higher heart radiation dose on risk of cardiovascular disease: A retrospective cohort study. International journal of surgery (London, England). 2018;53:345–9. Epub 2018/04/21. doi: 10.1016/j.ijsu.2018.04.018 . 29673690

[37] Fan C, Feng Q, Chen Y, Zhai Y, Zhou Z, Chen D, et al. Postoperative radiotherapy for completely resected Masaoka stage III thymoma: a retrospective study of 65 cases from a single institution. Radiation oncology (London, England). 2013;8:199. Epub 2013/08/14. doi: 10.1186/1748-717X-8-199 ; Central PMCID: PMC3751735. 23937886 PMC3751735

[38] Zhou D, Deng XF, Liu QX, Zheng H, Min JX, Dai JG. The Effectiveness of Postoperative Radiotherapy in Patients With Completely Resected Thymoma: A Meta-Analysis. The Annals of thoracic surgery. 2016;101(1):305–10. Epub 2015/09/14. doi: 10.1016/j.athoracsur.2015.06.034 . 26363651

[39] Lim YJ, Kim E, Kim HJ, Wu HG, Yan J, Liu Q, et al. Survival Impact of Adjuvant Radiation Therapy in Masaoka Stage II to IV Thymomas: A Systematic Review and Meta-analysis. International journal of radiation oncology, biology, physics. 2016;94(5):1129–36. Epub 2016/03/31. doi: 10.1016/j.ijrobp.2016.01.007 . 27026316

[40] Ma J, Sun X, Huang L, Xiong Z, Yuan M, Zhang S, et al. Postoperative radiotherapy and tumor recurrence after complete resection of stage II/III thymic tumor: a meta-analysis of cohort studies. OncoTargets and therapy. 2016;9:4517–26. Epub 2016/08/16. doi: 10.2147/OTT.S104435 ; PubMed Central PMCID: PMC4966637. 27524907 PMC4966637

[41] Girard N, Ruffini E, Marx A, Faivre-Finn C, Peters S. Thymic epithelial tumours: ESMO Clinical Practice Guidelines for diagnosis, treatment and follow-up. Annals of oncology: official journal of the European Society for Medical Oncology. 2015;26 Suppl 5:v40–55. Epub 2015/09/01. doi: 10.1093/annonc/mdv277 . 26314779

