# Supplementary material for: Evaluation of the role of postoperative radiotherapy in locally invasive thymoma: A propensity-matched study based on the SEER database
Source: PLoS One. 2023 Apr 13;18(4):e0283192. doi: 10.1371/journal.pone.0283192 (PMC10101529; doi:10.1371/journal.pone.0283192)
Supplement: S1 Table — (DOCX) [file pone.0283192.s001.docx]

S1 Table . Patient characteristics before and after propensity score matching with effect size

| **Variables** | **Before Propensity Score Matching** | | | **Effect Size**  **（SMD）** | **After Propensity Score Matching** | | | **Effect Size**  **（SMD）** |
| --- | --- | --- | --- | --- | --- | --- | --- | --- |
|  | **PORT (+)**  **(N = 420)** | **PORT (−)**  **(N = 280)** | **P** |  | **PORT (+)**  **(N = 262)** | **PORT (−)**  **(N = 262)** | **P** |  |
| Age |  |  |  |  |  |  |  |  |
| <40 | 47(11.2) | 32(11.4) | 0.009 | 0.139 | 25(9.5) | 29(11.1) | 0.19 | 0.108 |
| 40-49 | 69(16.4) | 41(14.6) |  |  | 33(12.6) | 38(14.5) |  |  |
| 50–59 | 106(25.2) | 48(17.2) |  |  | 64(24.4) | 46(17.5) |  |  |
| 60–69 | 117(27.9) | 75(26.8) |  |  | 80(30.6) | 72(27.5) |  |  |
| ≥70 | 81(19.3) | 84(30.0) |  |  | 60(22.9) | 77(29.4) |  |  |
| gender |  |  |  |  |  |  |  |  |
| Men | 219(52.1) | 130(46.4) | 0.139 | 0.056 | 127(48.5) | 123(46.9) | 0.726 | -0.150 |
| Women | 201(47.9) | 150(53.6) |  |  | 135(51.5) | 139(53.1) |  |  |
| Race |  |  |  |  |  |  |  |  |
| White | 282(67.2) | 188(67.1) | 0.804 | 0.072 | 181(69.1) | 182(69.5) | 0.587 | 0.045 |
| Black | 51(12.1) | 40(14.3) |  |  | 30(11.4) | 36(13.7) |  |  |
| Others | 79(18.8) | 47(16.8) |  |  | 51(19.5) | 44(16.8) |  |  |
| Unknown | 8(1.9) | 5(1.8) |  |  |  |  |  |  |
| Marital status |  |  |  |  |  |  |  |  |
| Married | 262(62.4) | 177(63.2) | 0.974 | 0.086 | 150(57.3) | 152(58.0) | 0.860 | 0.073 |
| Not married | 147(35.0) | 96(34.3) |  |  | 112(42.7) | 110(42.0) |  |  |
| Unknown | 11(2.6) | 7(2.5) |  |  |  |  |  |  |
| Tumor extent |  |  |  |  |  |  |  |  |
| Adjacent connective tissue | 176(41.9) | 117(41.8) | 0.975 | -0.001 | 129(49.2) | 111(42.4) | 0.115 | -0.069 |
| Adjacent organs or structures | 244(58.1) | 163(58.2) |  |  | 133(50.8) | 151(57.6) |  |  |
| Lymph node status |  |  |  |  |  |  |  |  |
| Negative | 379(90.3) | 254(90.7) | 0.978 | 0.008 | 247(94.3) | 252(96.2) | 0.305 | 0.045 |
| Positive | 19(4.5) | 12(4.3) |  |  | 15(5.7) | 10(3.8) |  |  |
| Unknown | 22(5.2) | 14(5.0) |  |  |  |  |  |  |
| Extent of surgery |  |  |  |  |  |  |  |  |
| Radical surgery | 119(28.3) | 70(25.0) | 0.047 | 0.107 | 69(26.3) | 64(24.5) | 0.157 | 0.100 |
| Total resection | 186(44.3) | 130(46.4) |  |  | 116(44.3) | 125(47.7) |  |  |
| Simple or partial resection | 97(23.1) | 77(27.5) |  |  | 66(25.2) | 70(26.7) |  |  |
| Debulking surgery | 18(4.3) | 3(1.1) |  |  | 11(4.2) | 3(1.1) |  |  |

Abbreviations: PORT, postoperative radiotherapy；SMD, standardized difference mean.
